# Metabolomic Reprogramming of C57BL/6-Macrophages during Early Infection with *L. amazonensis*

**DOI:** 10.3390/ijms22136883

**Published:** 2021-06-26

**Authors:** Maricruz Mamani-Huanca, Sandra Marcia Muxel, Stephanie Maia Acuña, Lucile Maria Floeter-Winter, Coral Barbas, Ángeles López-Gonzálvez

**Affiliations:** 1 Centre for Metabolomics and Bioanalysis (CEMBIO), Department of Chemistry and Biochemistry, Facultad de Farmacia, Universidad San Pablo-CEU, CEU Universities, Urbanización Montepríncipe, Boadilla del Monte, 28660 Madrid, Spain; mar.mamani.ce@ceindo.ceu.es (M.M.-H.); sandrammuxel@usp.br (S.M.M.); cbarbas@ceu.es (C.B.); 2 Departamento de Fisiologia, Instituto de Biociências, Universidade de São Paulo, Rua do Matão, Travessa 14, 101, São Paulo CEP-05508-090, SP, Brazil; stephanie.acuna@usp.br

**Keywords:** polyamines pathway, metabolites, bone marrow-derived macrophages, arginase, nitric oxide synthase

## Abstract

*Leishmania* survival inside macrophages depends on factors that lead to the immune response evasion during the infection. In this context, the metabolic scenario of the host cell–parasite relationship can be crucial to understanding how this parasite can survive inside host cells due to the host’s metabolic pathways reprogramming. In this work, we aimed to analyze metabolic networks of bone marrow-derived macrophages from C57BL/6 mice infected with *Leishmania amazonensis* wild type (*La*-WT) or arginase knocked out (*La*-arg^−^), using the untargeted Capillary Electrophoresis-Mass Spectrometry (CE-MS) approach to assess metabolomic profile. Macrophages showed specific changes in metabolite abundance upon *Leishmania* infection, as well as in the absence of parasite-arginase. The absence of *L. amazonensis*-arginase promoted the regulation of both host and parasite urea cycle, glycine and serine metabolism, ammonia recycling, metabolism of arginine, proline, aspartate, glutamate, spermidine, spermine, methylhistidine, and glutathione metabolism. The increased L-arginine, L-citrulline, L-glutamine, oxidized glutathione, S-adenosylmethionine, *N*-acetylspermidine, trypanothione disulfide, and trypanothione levels were observed in *La*-WT-infected C57BL/6-macrophage compared to uninfected. The absence of parasite arginase increased L-arginine, argininic acid, and citrulline levels and reduced ornithine, putrescine, S-adenosylmethionine, glutamic acid, proline, *N*-glutamyl-alanine, glutamyl-arginine, trypanothione disulfide, and trypanothione when compared to *La*-WT infected macrophage. Moreover, the absence of parasite arginase leads to an increase in NO production levels and a higher infectivity rate at 4 h of infection. The data presented here show a host-dependent regulation of metabolomic profiles of C57BL/6 macrophages compared to the previously observed BALB/c macrophages infected with *L. amazonensis,* an important fact due to the dual and contrasting macrophage phenotypes of those mice. In addition, the *Leishmania*-arginase showed interference with the urea cycle, glycine, and glutathione metabolism during host–pathogen interactions.

## 1. Introduction

Macrophages are a heterogeneous population of cells that can acquire distinct functional phenotypes in response to cytokines or microbial antigens, playing a critical role in innate and adaptative immune responses [1,2]. Macrophages have a broad spectrum of functional phenotypes, classically represented by two extreme polarizations, pro-inflammatory M1 macrophages presenting microbicidal activity and anti-inflammatory M2 macrophages with activity in tissue repair, wound healing, and in response against parasitic infections [3]. M1 macrophages are induced by recognition of microbial antigens, such as lipopolysaccharide (LPS) and lipophosphoglycan (LPG), via pattern recognition receptors (PRRs) as Toll-like receptors (TLRs) and by pro-inflammatory cytokines as interferon-gamma (IFN-γ) and tumor necrosis factor-alpha (TNF-α) [4,5,6]. They can also be characterized by the expression of nitric oxide synthase 2 (NOS2), producing microbicidal agent nitric oxide (NO) from L-arginine. The production of these free radical blocks, commonly the oxidative phosphorylation (OXPHOS), leads to a glycolytic phenotype upon the ATP production [7,8,9]. Additionally, the NOS2 activity is dependent on four co-factors, the cytosolic NADPH being the first [10]. To support the NO production, M1 macrophages present a high flux through the pentose phosphate pathway (PPP), generating the necessary NADPH [11,12,13]. They also show an increase in particular tricarboxylic cycle (TCA cycle) metabolites such as citrate, succinate, and itaconate [14,15]. Moreover, M2 macrophages express arginase-1 (ARG1) which uses arginine to increase ornithine, a substrate to polyamines production (putrescine, spermidine, and spermine). These molecules are used by cells to proliferate, synthesize collagen, and promote tissue remodeling [16,17]. In a metabolic approach, M2 macrophages have enhanced OXPHOS and fatty acid oxidation [7,8,9]; sometimes, they can appear foamy, being strongly related to the severity of atherosclerosis to the accumulation of cholesterol [18,19].

A combination of stimuli usually determines macrophages phenotypes. They can recognize many pathogens, such as *Leishmania amazonensis*, an intracellular parasite transmitted to mammals via the bite of a sand fly vector. The infection caused by this protozoan leads to tegumentary leishmaniasis in humans [20,21]. On the other hand, during the early phase of mice macrophage infection, the parasite promotes a modulation in host-gene expression, distinctly in BALB/c and C57BL/6, implicating in the metabolism of arginine and, consequently, implicating balance in M1/M2 macrophages during in vitro infection [22,23,24]. BALB/c-macrophages are more susceptible to late in vitro infection than C57BL/6-macrophages, but infectivity in the early phase is similar [22,23,24]. Additionally, in vivo infections show that BALB/c mice are susceptible to *L. amazonensis* infection, presenting severe cutaneous lesions, whereas C57BL/6 mice present a moderate lesion size [25,26].

The L-arginine metabolism balances the macrophages leishmanicidal activity and parasite survival [24,27,28,29,30]. *Leishmania* arginase enzyme is essential for parasite survival and infectivity [31]. Promastigote forms of *L. amazonensis* knockout for arginase (*La*-arg^−^) present a change in the metabolite content as a result of the absence of the enzyme, increasing the proline and glutamate levels to supply ornithine and putrescine, indicating that arginase activity is important to parasite survival [31,32,33]. Lower levels of ornithine and putrescine contrasting with higher arginine and citrulline levels are present promastigotes of *La*-arg^−^ compared to *L. amazonensis* wild-type (*La*-WT). The arginase from the parasite competes to arginine inside the macrophage, interfering in the gene expression and metabolism of host cells [34,35]. The modulation of arginine transporters can control the arginine availability on parasite and host cells [36,37,38,39,40].

The metabolome profile of BALB/c-macrophages indicated that the L-arginine metabolism favors polyamines production [24]. Here, we explore the metabolome fingerprint of C57BL/6-macrophages during early infection with *L. amazonensis*, using untargeted Capillary Electrophoresis-Mass Spectrometry (CE-MS) approach. The *L. amazonensis* infection alters the mix of host and parasite metabolites from the urea cycle, glycine, and serine metabolism, ammonia recycling, arginine metabolism, proline, aspartate, glutamate, spermidine, histidine, and glutathione metabolism. The absence of parasite arginase implicates in the increased levels of arginine, NO, polyamines production, glutathione and trypanothione, and parasite arginase function on macrophage metabolism.

## 2. Results

### 2.1. The Parasite Arginase Modulates NO Production and Infectivity in Leishmania-Infected Macrophages

**Figure 1:**
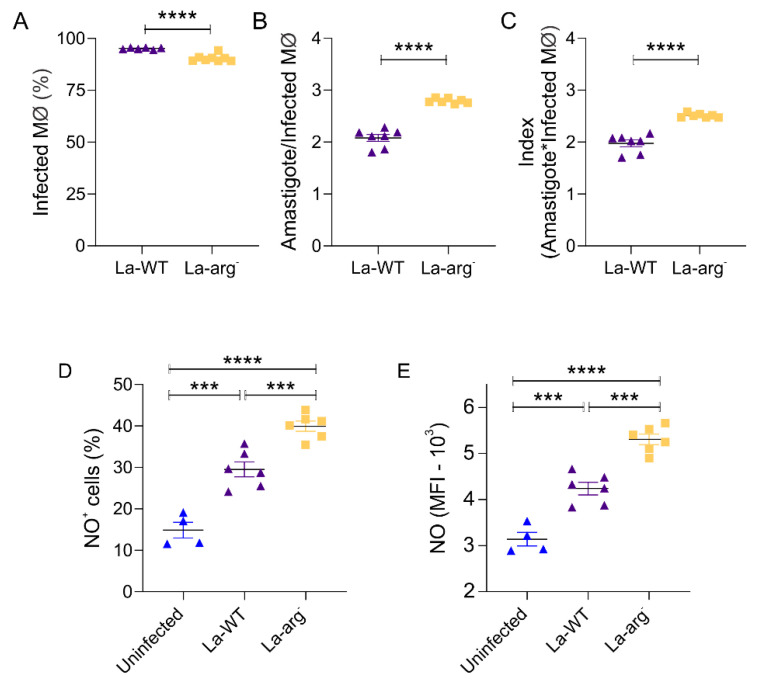
Infection index and NO production in C57BL/6 macrophages infected with *L. amazonensis La*-WT or *La*-arg^−^. (**A**) C57BL/6-macrophages (5 × 10^5^) were infected with CFSE-labelled *L. amazonensis* wild type (purple triangle) or with *L. amazonensis* arginase knockout (yellow square) (MOI5:1) and collected after 4 h for analysis of infectivity by image flow cytometer, gating in the CFSE-internalized cells to count the frequency of infected macrophages (infected MØ). (**B**) Spot count tool to determine the number of infected MØ. (**C**) Index of infection was calculated by multiplying the rate of infected MØ by the number of amastigotes per infected MØ. (**D**) C57BL/6-macrophages (5 × 10^5^) were infected with *L. amazonensis* wild type (purple triangle) or with *L. amazonensis* arginase knockout (yellow square) (MOI5:1), and after 4 h were labeled with 5 μM of DAF-FM for NO quantification in image flow cytometry, gating in DAF-FM+ cell quantify the frequency of NO producing cells (NO^+^ cells). (**E**) Media of fluorescence intensity (MFI). The values were compared to the non-infected macrophages (blue triangle). Each point represents the individual values (n = 6). (***) *p-*value < 0.01; (****) *p-*value < 0.001.

The infectivity of *La*-WT or *La*-arg^−^ in C57BL/6 macrophages were analysed after 4, 24, 48, or 72 h of infection. The frequency of infected macrophages, the number of amastigotes per infected macrophages, and index of infection (rate of infected macrophages multiplied by the number of amastigotes per infected macrophages) were performed in image flow cytometry. The frequency of infected macrophages was reduced in C57BL/6-*La*-arg^−^ (90.46% ± 0.60) compared to C57BL/6-*La*-WT (95.20% ± 0.19) at 4 h of infection, but the number of amastigotes per infected macrophages was increased in C57BL/6-*La*-arg^−^ (2.79 ± 0.06) compared to C57Bl/6-*La*-WT (2.07 ± 0.01) at 4 h of infection (Figure 1A–C). Additionally, after 24 and 48 h of infection, the number of amastigotes per infected macrophages was higher in C57BL/6-*La*-arg^−^ than C57BL/6-*La*-WT, but showed a time-coursed stabilization. Consequently, the infection index was higher in C57BL/6-*La*-arg^−^ than to C57BL/6-*La*-WT at 4, 24, and 48 h of infection (Supplementary Figure S1). See Supplementary Figure S2 for details of Infectivity Analysis Gate Strategy.

To evaluate the ability of C57BL/6 macrophages infected with *L. amazonensis* to produce NO and the impact of parasite arginase, macrophages uninfected and infected for 4 h with *L. amazonensis La*-WT or with *La*-arg^−^ were labeled with DAF-FM^+^, and the frequency of DAF-FM^+^ cells and MFI were analyzed in image flow cytometry (Figure 1D,E). After 4 h, the frequency of cells producing NO and MFI in C57BL/6-*La*-WT was increased 1.98-times and 1.35-times, respectively, in relation to those uninfected. Additionally, in the infection with *La-*arg^−^, the frequency of cells producing NO increased by 2.68-times, and MFI 1.69-times, in relation to uninfected. The frequency of cells producing NO was 1.35-times, and MFI was 1.25 high in C57BL/6-*La*-arg^−^ compared with C57BL/6-*La*-WT. These data indicated a differential NO-production regulation during infection with *L. amazonensis* influenced by parasite arginase activity. See Supplementary Figure S3 for details of NO Quantification Gates Strategy.

### 2.2. Statistical Analyses of the Metabolic Profile of Leishmania Infected C57BL/6-Macrophages

Three groups of samples from C57BL/6 macrophages infected with *L. amazonensis* (*La*-WT) (n = 9), C57BL/6 macrophages infected with *L. amazonensis* knockout for arginase (*La*-arg^−^) (n = 10), and uninfected C57BL/6 macrophages (n = 10) were used for the untargeted metabolomics CE-MS approach.

**Figure 2:**
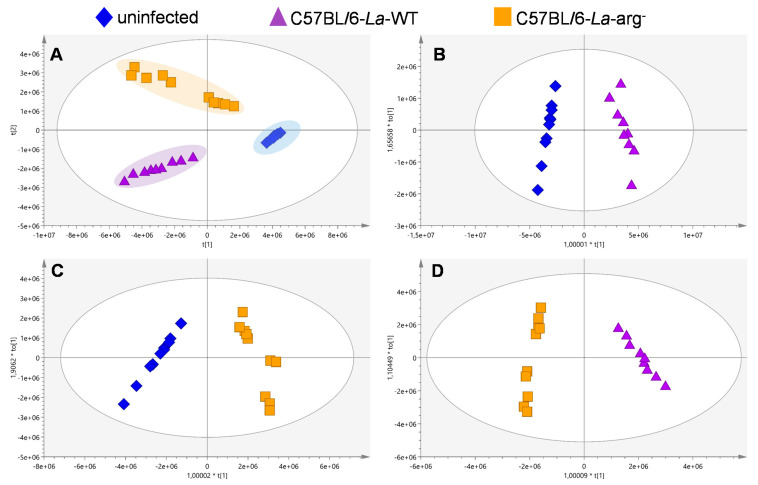
Partial least squares discriminant analysis (PLS-DA) and Orthogonal partial least-squares-discriminant analysis (OPLS-DA) analysis of variation between the two groups of *L. amazonensis* infected C57BL/6-macrophages. (**A**) Score plots for the PLS-DA and model were built to compare three conditions: C57BL/6-*La*-arg^−^ infected macrophage, C57BL/6-*La*-WT infected macrophage, and uninfected macrophage (R^2^ = 0.957 and Q^2^ = 0.934). OPLS-DA model was built for the comparison; (**B**) C57BL/6-*La*-WT infected macrophage vs. uninfected macrophage (R^2^ = 0.975 and Q^2^ = 0.965); (**C**) C57BL/6-*La*-arg^−^-infected macrophage vs. uninfected macrophage (R^2^ = 0.914 and Q^2^ = 0.901); and (**D**) C57BL/6-*La*-arg^−^ infected macrophage vs. C57BL/6-*La*-WT infected macrophage (R^2^ = 0.957 and Q^2^ = 0.947).

As a result of sample analysis and data processing, a total of 269 features were obtained after the deconvolution. The quality of the analysis was evaluated along with the experiment by observing the quality control (QC) clustering in the unsupervised PCA-X plot model (Supplementary Figure S4). PCA-X plot model showed R^2^ = 0.991 indicating good quality of sample analysis. The model was also built to analyze PLS-DA discrimination. The clustering of the three different groups is observed, indicating that the metabolites’ levels have changed due to the macrophages’ infection. These differences were evaluated by an OPLS-DA model, concerning the quality of the multivariate models obtained; all the models presented good quality, presenting R^2^ ≥ 0.957 and Q^2^ ≥ 0.947 values (Figure 2). Confidence intervals of Jack-Knife, correlation *p* (*p*corr) > |0.5|, and variables importance in projection (VIP) >1 were calculated to identify the features that are statistically significant in the multivariate (MVA) statistics (Supplementary Table S1).

The univariate analysis (UVA) statistics results using non-parametric one-way ANOVA (Kruskal–Wallis test) showed a greater number of significant variables. Consequently, pairs of groups were compared, and those with a *p* < 0.05 were considered significant metabolites using the Mann–Whitney U test. These features were annotated by monosotopic mass and relative migration time (RMT) using an in-house library available at CEUMassMediator (CE-MS search), an online open-source tool developed in our laboratory (http://ceumass.eps.uspceu.es/index_cesearch.xhtml) (May 2021) [41]. Fifty-three metabolites were found to be statistically significant, revealing the modulation of infection. Fifty-two metabolites appeared to be modulated in *La*-WT-infected compared to non-infected macrophages, indicating that infection with *L. amazonensis* results in an increased abundance of metabolites mediated by its metabolism within macrophages and/or manipulate the metabolism of the host. Forty-seven metabolites appeared modified in macrophages infected with *La*-arg^−^ compared to the non-infected ones, as a result of the metabolism of the parasite in the host cell and the activation of microbicides mechanisms in macrophages (Supplementary Table S1). To observe parasite arginase activity inside the host cell, the *La*-arg^−^ and *La*-WT-infected C57BL/6-macrophages were compared (Table 1), revealing 34 modulated metabolites.

### 2.3. Parasite Arginase Activity Leads to Differential Metabolites Profiles of C57BL/6-Macrophages Infected with L. amazonensis

**Figure 3:**
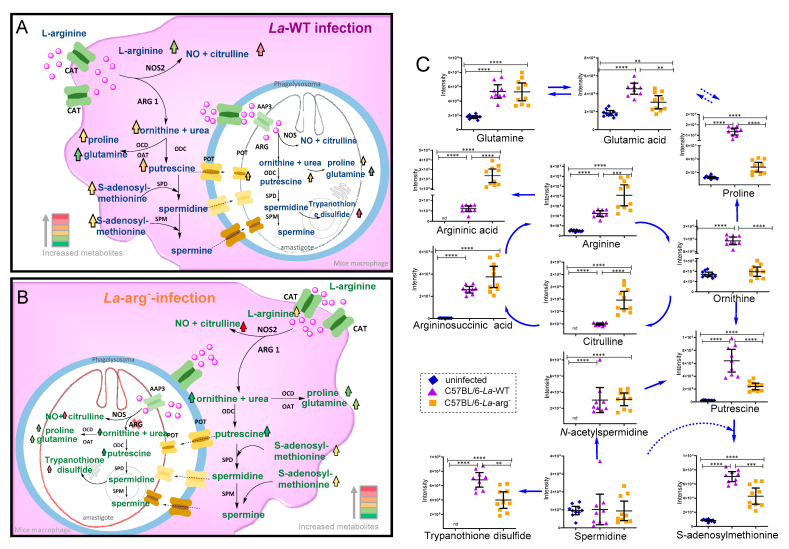
Schematic representation of L-arginine metabolism in *Leishmania*-infected macrophages. (**A**) *L. amazonensis* WT infection can increase the levels of L-arginine via uptake by cationic amino acid transporter (CAT) in macrophage, and via amino acid permease 3 (AAP3) in amastigote form of *Leishmania*. L-arginine can be converted by host nitric oxide synthase 2 in nitric oxide and citrulline, as seen at its increased levels, leading a leishmanicidal activity or parasite-NOS, regulating amastigote survival and replication. On the other hand, host arginase 1 (ARG1) and parasite arginase (ARG) competes by arginine to produce ornithine and subsequently in polyamines: putrescine via ornithine decarboxylase (ODC), which is used to produce spermidine via spermidine synthase (SPD) and subsequently used by spermine synthase (SPM) to produce spermidine, using S-adenosylmethione as substrate. Ornithine can be used by ornithine decarboxylase (OCD) to produce proline or by ornithine aminotransferase (OAT) to produce glutamine. (**B**) The absence of parasite arginase in *La*-arg^−^-infected macrophages increased arginine availability, production of higher levels of citrulline, and allowed a slight increase in ornithine production, altering the levels of polyamines and trypanothione disulfide. Red color indicates increased levels of metabolites, and green indicates reduced levels of metabolites. nd – not determined (**C**) Using an untargeted CE-MS, the intensity peak area of metabolites from the metabolic profile of macrophages infected with *La*-WT or *La*-arg- and uninfected were compared, focusing on the L-arginine metabolism pathways. The graphs are representing the average metabolite amount in each group. **) *p*-value < 0.01; (***) *p*-value < 0.005; (****) *p*-value < 0.001,was used non-parametric 1-way ANOVA test (Kruskal–Wallis).

**Figure 4:**
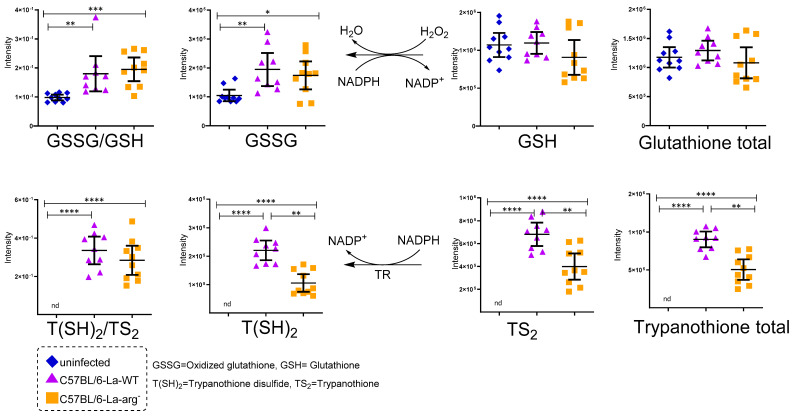
Antioxidant systems glutathione and trypanothione. Total glutathione (GSH + GSSG), oxidative stress (GSSH/GSH ratio), total trypanothione (T(SH)_2_ + TS_2_) and T(SH)_2_/TS_2_ ratio were compared in macrophages infected with *La*-WT or *La*-arg- and uninfected. (*) *p*-value < 0.05; (**) *p*-value < 0.01; (***) *p*-value < 0.005 (****) *p*-value < 0.001, was used non-parametric 1-way ANOVA test (Kruskal–Wallis).

The overview of the untargeted metabolome of *La*-WT-infected macrophages or *La*-arg^−^-infected macrophages compared to uninfected macrophages showed an increased level of arginine, argininic acid, citrulline, arginosuccinic acid, ornithine, putrescine, *N*-acetylspermidine, S-adenosylmethionine, oxidized glutathione, trypanothione disulfide, proline, glutamine, and glutamic acid, as observed by their percentage change in Table S1 and intensity of peak area of each metabolite in Figure 3C and Figure 4. The variations of the predictors correlated and orthogonal to each group’s response were evaluated in Figure 2B,C.

Focusing on investigating the parasite arginase impacts on the metabolism of infected macrophages, we compared the levels of metabolites from *La*-arg^−^-infected macrophages versus *La*-WT-infected macrophages (Table 1 and Figure 3 and Figure 4). Based on metabolites from L-arginine metabolism, the absence of parasite arginase upregulated arginine, citrulline, and argininic acid levels. On the other hand, it downregulated the levels of putrescine, proline, ornithine, glutamic acid, S-adenosylmethionine, cystathionine, *N*-carbamyl- arginine, trypanothione disulfide, and trypanothione.

**Figure 5:**
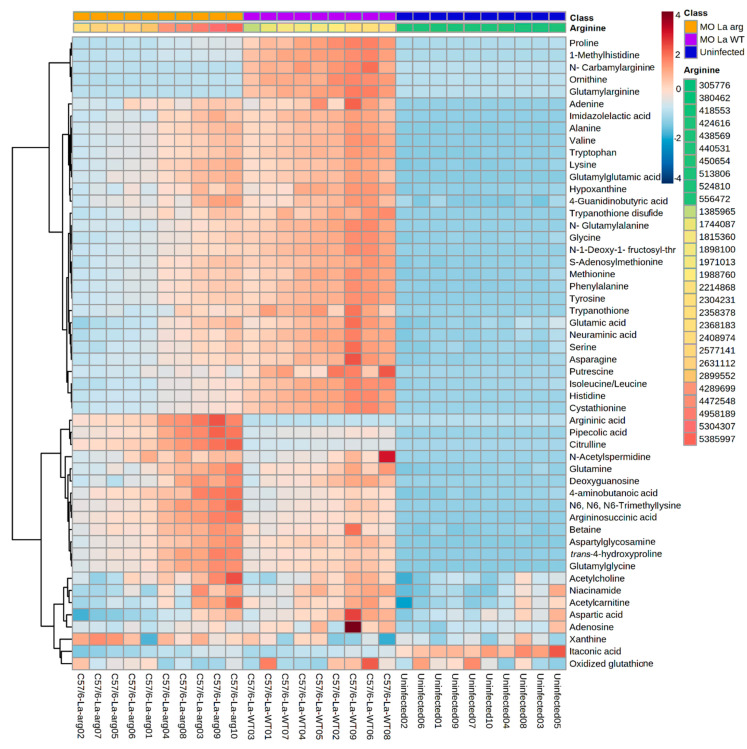
Heatmap of 53 metabolites differentially modulated in the metabolomic analysis of macrophages infected with *La*-WT or *La*-arg^−^ versus uninfected macrophages. Each column represents a sample, and each row represents a metabolite. Two factors order the samples: the first is arranged in three main groups (Phenotype: uninfected, *La*-arg^−^ and *La*-WT), and inside each one, samples are ordered by the abundance of arginine, that has a pseudo-color ranging from the lowest amount–green; to the highest–red. The color code inside the heatmap depicts each metabolite relative fold change between groups. The parameters used for the analysis were the Euclidean distance measure and the Ward cluster algorithm.

Furthermore, heatmap analysis of the magnitude, directionality, and significance for the association of the 53 differentially modulated metabolites showed the clustering of *La*-arg^−^-infected macrophages and *La*-WT-infected macrophages in a distinct way of uninfected macrophages (Figure 5).

## 3. Discussion

Macrophage activation and functional plasticity are strictly connected to metabolic reprogramming, supplying energy and metabolite participation in anabolic and catabolic pathways, modifying cell response to infections. Macrophages alter the demands for glucose, fatty acids, amino acids, purines, ATP, and NADPH to allow an appropriate response against pathogens, avoiding excessive inflammation or tissue injury [42,43].

Additionally, *Leishmania* can regulate its metabolites content during the differentiation from promastigote to amastigote inside macrophages. They can also evade microbicidal mechanisms [44,45]. *Leishmania* is auxotrophic for many nutrients that the amastigote can uptake from phagolysosomes, such as amino acids, lipids, purines, pyrimidines, heme, and vitamins [34,46,47]. The metabolite uptake and de novo synthesis correlate with virulence maintenance and parasite survival and can be favored by phagolysosome niche [34,46,47]. Many studies have shown that the metabolism from parasites interfere in macrophage metabolisms, but this is still poorly understood. However, to fill this gap, our group has previously demonstrated the influence of *L. amazonensis* arginase in the metabolism of promastigotes forms, changing the arginine, glutamine, and proline profile, as well as interfering in polyamines biosynthesis, as revealed by CE-ESI(+)-MS strategy [33]. Additionally, the changes in arginine levels and metabolism were shown in *L. braziliensis* resistant to antimony treatment, through CE-ESI(+)-MS strategy [33], evidencing alterations in the amino acids content and metabolism, and thiol-dependent redox [48].

Clinical data of diffused cutaneous leishmaniasis (DCL) showed that patients present higher ornithine, spermidine, and citrulline in plasma samples than mucocutaneous leishmaniasis (MCL), using HPLC tool [49]. Indeed, DCL skin biopsies upregulated arginase 1 (ARG1) and spermine synthase mRNAs involved in polyamines biosynthesis concerning localized cutaneous leishmaniasis (LC) and MCL lesions, highlighting the significance of host ARG1 and arginine transporter (cationic amino acid transporter, CAT2) in MCL [49]. However, the ARG1 and arginase 2 (ARG2) transcripts are lower in DCL than healthy controls [49]. *L. amazonensis* infection of BALB/c-macrophages increases host *Cat*2 and *Arg*1 transcripts, parasite arginine transporter (amino acid permease 3, AAP3), and parasite arginase [23]. Despite this, C57BL/6-macrophages infected with *L. amazonensis* did not modulate those genes [28,50].

Additionally, the evaluation of metabolome fingerprint of early infection of *L. amazonensis* BALB/c infected macrophages by CE-ESI(+)M showed a modified blend of metabolite content, changing the amino acids, polyamines, and purine pathways [24]. Similar to the observed BALB/c macrophages infected with *L. amazonensis* [24], C57BL/6-macrophages also showed increased L-arginine levels, ornithine, and putrescine, supporting the metabolization of L-arginine in polyamines, which supply the production of trypanothione in detriment of glutathione.

However, L-arginine is used to produce polyamines, and is necessary to access the main M1 character: NO production. This production is dependent on the availability of the amino acid, O_2_ plus the co-factors: reduced nicotinamide-adenine-dinucleotide phosphate (NADPH), flavin adenine dinucleotide (FAD, flavin mononucleotide (FMN), and tetrahydrobiopterin (BH_4_). Unlike the constitutive forms, NOS2 depends on calmodulin and heme to form a functional homodimer [11,12,51]. Contrasting with *L. amazonensis* infection of BALB/c macrophages that presents lower levels of NOS2, NO, and citrulline [22,23,24], the increased levels of citrulline in C57BL/6-macrophages infected with *L. amazonensis* compared to uninfected ones support the high levels of NO production by NOS2 and the reduction in infected macrophages in the time-course of infection. Additionally, a low levels of amastigotes inside C57BL/6-macrophages, as observed in the time-course of in vitro infection (Supplementary Figure S1) can be seem, in contrast to observed in susceptible BALB/c macrophage [24]. The absence of parasite arginase had no effect on the number of amastigotes per macrophages at 4–48 h, suggesting parasite were not multiplying. The higher levels of frequency of NO producing cells increased the MFI of DAF-FM labeling per cell, and citrulline in C57BL/6 macrophages infected with *La*-arg^−^ indicate a microbicidal macrophages phenotype in this condition. It is interesting to note that the correlation of ARG1/NOS2 ratio and increase in susceptibility to *Leishmania* infection is extensively studied [52], but more information can be explored in the relation of polyamines/NO production. However, polyamines production can reduce NO production, as they are cross-inhibiting pathways. Polyamines are polycationic metabolites crucial for cell proliferation, gene transcription, protein translation, oxidative stress regulation, and immunosuppression signals [44,53,54,55]. Their biosynthesis also depend on arginine content competing with NO production [29,56,57]. The intricate cascade of enzymes that use L-arginine and their substrates or products and transporters allows the integration of polyamines biosynthesis with other metabolites, modulating the immune response [58,59]. Furthermore, increased ornithine levels, a product of ARG1, in C57BL/6 macrophages infected with *L. amazonensis* can be converted in proline, as indicated by its higher levels suggesting ornithine cyclodeaminase (OCD) activity (Figure 3A,C) [60,61]. Ornithine and proline can supply the increased levels of glutamate and glutamine in a complex enzyme network [59,62] involving the production of metabolites: L-1-pyrroline 5-carboxylate, L-glutamate 5-semialdehyde, and L-glutamyl-P, [44] which are characterized in macrophages metabolism but are not identified by our method of analysis.

Further, we found the same spermidine levels between *La*-WT infected and uninfected macrophages, but higher S-adenosylmethionine and *N*-acetylspermidine levels in infected macrophages (Figure 3A,C). S-adenosylmethionine donates an aminopropyl group to spermidine and spermine guided by aminopropyl transferases called spermidine synthase and spermine synthase [59,62]. The S-acetylspermidine is a polyamine with an acetyl group at the spermidine *N*_1_-position received by spermidine/spermine *N*_1_-acetyltransferase (SSAT) [63]. The mammalian cell polyamine content is maintained by enzymes that have a crucial role to this [64,65,66], we can exemplify: a) ARG1; b) OODC; c) SSAT; d) APAO (acetyl polyamine oxidase—which converts *N*_1_-acetylspermidine into putrescine plus *N*-acetyl-3-aminopropanaldehyde [67]; and e) SMO (spermine oxidase—which converts spermine to spermidine plus 3-aminopropanaldehyde) [68,69]. Although found in mammalian cells, an APO and SMO [70] in *Leishmania* must be confirmed.

Another role of polyamines in the control of translation, mediated by spermidine, is an essential activity in the activation of eukaryotic initiation factor 5A (eIF5A), adding a hypusine through a process that cleaves the spermidine and transfer of 4-aminobutyl moiety to an internal lysine to form an intermediate deoxyhypusine and subsequently hypusine residue and mature eIF5A, being a post-translational modification. This protein modification is required to synthesize proteins in host cells [55,71,72] and *Leishmania* [73].

Regarding the redox state control, L-arginine serves as a glutathione source, which has particular relevance when detoxifying the cell from mitochondrial oxidants, regulating the redox environment [62]. The relative increase in oxidized glutathione (glutathione disulfide, GSSG)/reduced glutathione (glutathione, GSH) ratio observed in *La*-WT and *La*-arg^−^ infected macrophages versus uninfected macrophages may indicate this response led to the oxidative stress condition (Figure 4). Peroxides oxidize GSH glutathione in a reaction catalyzed by glutathione peroxidase (GPx), ligating two reduced glutathione (GSH) with a disulfide bond, forming the GSSG. This reaction diminishes peroxide levels, removing these oxidants [74,75]. The turnover is mediated by the reduction in GSSG to GSH using NADPH as an electron donor. Glutathione reductase (GR) mediates this reaction. Additionally, glutathione biosynthesis is involved in resistance to anti-leishmanicidal agent pentavalent antimonial (Sb V) [76]. The fine-tuning integration of oxidative stress and polyamines biosynthesis may balance the parasite killing in macrophages.

The integration of those pathways can be seen by analyzing the level of S-adenosylmethionine that is increased in the *La-*arg^−^ infected macrophages in relation to non-infected macrophages but decreased in relation to the *La*-WT infected macrophages, although the levels of glutathione were similar in any of these macrophages. The decrease in S-adenosylmethionine in *La*-arg^−^ infected macrophages could be explained as a compensatory effect as it should be used to produce glutathione as well as to supply the production of putrescine, spermine, and spermidine as these molecules can be taken by the parasite to produce trypanothione, as parasite ornithine-putrescine-spermine-spermidine pathway is harmed by the absence of arginase activity. However, this possible compensation is not enough to reach the same levels of trypanothione in the La-*arg*^−^ infected macrophages compared to the detected in WT infected macrophages.

**Figure 6:**
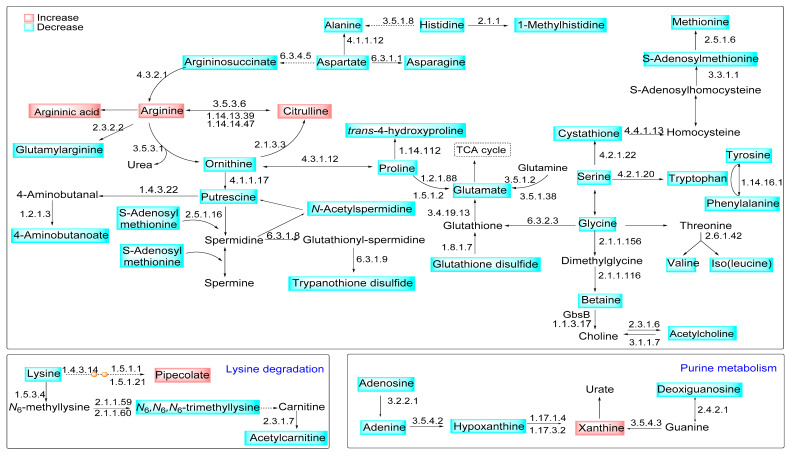
Modulation of metabolite content in the L-arginine metabolic pathways in macrophages infected with *La*-arg^−^ compared to *La*-WT. Representation of modulated metabolic pathways during the infection of macrophages with *L. amazonensis*. The blue boxes represent the decreased metabolites, while the red boxes represent the increased metabolites in the comparison of *La*-arg^−^ to *La*-WT. The KEGG EC entry of enzymes involved in the pathway is shown as a number close to the arrows, indicating the catalyzed reactions.

In fact, trypanosomatids do not show glutathione, but an analogous: trypanothione, formed by conjugation of spermidine and glutathione by trypanothione synthetase amidase (TRYS), unique in *T. brucei*, *T. cruzi*, and *Leishmania* spp. [77,78,79,80,81,82], and presenting a crucial function in combat oxidative stress [83,84] and resistance to anti-trypanosome drugs [79,85,86]. This molecule is also related to L-arginine metabolism. We also found higher oxidized trypanothione (trypanothione disulphide, T(SH_2_))/reduced trypanothione (trypanothione, TS_2_) ratio in macrophages infected with *La*-WT compared to *La*-arg^−^ or uninfected macrophages, probably impacting on the redox state (Figure 4). We suggest that this can also influence in the macrophage killing activity [62,87,88]. Similarly to glutathione, trypanothione needs to be recycled, depending on the NADPH availability produced by the pentose phosphate pathway (PPP) and the balance of competition between NADPH electron donors for NOS2 to produce NO, and NADPH oxidase to produce ROS and trypanothione reductase [87,88,89,90]. Notably, the higher levels of NO in *La*-arg^—^infected compared to *La*-WT-infected macrophages appeared in accord with higher levels of xanthine, once NO can help to inactive xanthine dehydrogenase and xanthine oxidase (interconvertible forms of xanthine oxidoreductase), increasing xanthine and decreasing ROS production and assisting in balancing damaging and helpful outcomes [91,92]. Another antioxidant molecule involved in protecting both host and parasite against oxidative stress is pipecolate, related to proline-ornithine metabolism, found in higher levels in *La*-arg^−^-infected compared to *La*-WT-infected macrophages (Table 1 and Figure 6) [82].

In accordance, the arginase absence influences glutamate and proline metabolism. It increases polyamines production and arginine, citrulline, and argininic acid levels. It also decreases putrescine, proline, ornithine, glutamic acid, S-adenosylmethionine, cystathionine, and *N*-carbamylarginine levels (Table 1 and Figure 6). Despite that, in a previous study, we did not find a modulation of the transcript levels involved in these metabolic pathways in the *L. amazonensis* infection context, comparing macrophages infected with *La*-WT and *La*-arg^−^ [50].

## 4. Materials and Methods

### 4.1. In Vitro Macrophage Infections

The Bone Marrow Derived Macrophages (BMDM) were seeded into 24-well plates (SPL Lifescience, Pocheon, Korea) (5 × 10^5^/SPL) for infectivity and NO production analysis, or into 6-well plates (SSPL Lifescience, Pocheon, Korea) (5 × 10^6^/well) for metabolite analysis. After 18 h of incubation at 34 °C in an atmosphere of 5% CO_2_, BMDMs were infected with *La*-WT or *La*-arg^−^ promastigotes in the stationary growth phase (MOI 5:1). After 4 h of infection, non-phagocyte promastigotes were washed with fresh medium, and samples were collected for metabolite extraction or fixed for infectivity index determination. The uninfected macrophages were maintained in the same conditions. (See Supplementary Information for details of macrophage harvest).

### 4.2. Infectivity Quantification

For infectivity analysis, before infection *La*-WT or La-arg^−^ promastigotes were labeled with 5 µM Carboxyfluorescein succinimidyl ester (CFSE) (Invitrogen) for 30 min and washed in RPMI supplemented as previously described (see Supporting Information for details of parasite culture). The infectivity was analyzed by Image Flow Cytometry (Amnis – Amnis Corporation, Seattle, WA, USA) after cell-fixation with 1% paraformaldehyde (Merck, Darmstadt, Germany) for 20 min at 4 °C, followed by PBS washing. Infectivity was analyzed in Spot Count wizard (ideas Software, Amnis), single gating cells, and CFSE^+^ population (infected macrophages) to count the number of amastigotes per infected/macrophage in at least 80,000 macrophages in three independent experiments. The infection index was calculated by multiplying the macrophage infection rate by the mean number of amastigotes per macrophage.

### 4.3. NO Quantification

For NO quantification, after 4 h of infection, macrophages were ungripped by incubation with 1 mM EDTA in PBS 1X for 10 min at 34 °C and cell scraping on ice after adding RPMI plus 10% FBS. Cells were centrifuged (500× *g*, 10 min, 4 °C) and FBS was resuspended in 50 μL of 5 μM of Diaminofluorescein-FM Diacetate (DAF-FM). After 30 min of incubation, cells were washed and resuspended in cold PBS 1X. NO was quantified by image flow cytometry, gating the frequency of DAF-FM^+^ cell and the media MFI of DAF-FM.

### 4.4. CE-MS Metabolic Fingerprinting

Sample preparation was performed following a protocol previously described (see Supplementary Information for details of Metabolite Extraction) [33]. The extracts obtained were analyzed by capillary electrophoresis system (7100 Agilent Technologies, Santa Clara, CA, USA) coupled to a time-of-flight mass spectrometer (6224 Agilent Technologies), the coupling was equipped with an ESI sprayer G1607 from Agilent Technologies. The compounds were separated in a fused silica capillary (Agilent Technologies) with a total length of 100 cm and an internal diameter of 50 μm in normal polarity with a background electrolyte (BGE) containing 1.0 M formic acid solution in 10% methanol (*v*/*v*). New capillaries were pre-conditioned with a flush (950 mbars) of NaOH 1.0 M for 30 min, followed by MilliQ water for 30 min and BGE for 30 min. Before each analysis, the capillaries were conditioned with BGE for 5 min. Samples were hydro-dynamically injected at 50 mbar for 50 s, and stacking was carried out, applying BGE at 100 mbar for 10 s. Metabolite separation occurred when a voltage of 30 kV with 25 mbar of internal pressure was applied. The observed current during the experiment under these conditions was 23 μA.

The sheath liquid flow composition was methanol/water 50:50 (*v*/*v*) with two reference standards, purine (C_5_H_4_N_4_, *m*/*z* 121.0509) and HP-0921 (C_18_H_18_O_6_N_3_P_3_F_24_, *m*/*z* 922.0098), for mass accuracy monitoring was supplied by ISO Pump 1200 from Agilent Technologies Santa Clara, CA, USA. After the separated sample compounds leave the capillary, a positively charged spray is formed by a flow of the sheath liquid (6 µL min^−1^), a nitrogen spraying pressure of 10 psi, and a capillary voltage of 3500 V. The spray is dried with a hot nitrogen flow of 10 mL min^−1^ at 200 °C. All ions formed in the gaseous state are directed to the TOF-MS following the parameter voltages: fragmentor 125 V, skimmer 65V, and octopole 750V. Data were collected in the positive ionization (+) mode of ESI, and ions between 70 and 1000 Da were acquired at a scan rate of 1.41 scans per second. All systems were controlled by Agilent MassHunter TOF data acquisition software version B.06.01 (Agilent Technologies Inc.).

### 4.5. CE-MS Data Treatment

Data acquired after CE-MS analysis were processed using MassHunter Profinder (B.10.00, Agilent Technologies Santa Clara, CA, USA) to obtain a data matrix. The software first performed an alignment and correlation of all electropherograms migration time using a specific algorithm for CE. Then, the software applies two consecutive algorithms to perform the deconvolution. First, it performs the extraction of the critical variables (compounds) and reduces the data by removing the unspecified information. A second algorithm improves the reliability of the search for data features. The Find by Ion (FbI) algorithm performs a targeted extraction of specific features. The abundance of the molecule, mass accuracy, and migration time of each feature in all samples was finally obtained as matrix data. Data quality was ensured by excluding background noise and non-related ions, the characteristics present in the QC were maintained at 50%. For missing values, the k-nearest neighbors (kNN) algorithm was applied. Data was re-filtered on relative standard deviation (RSD in the QC features were removed from all samples with an RSD 30%).

### 4.6. Statistical Analysis

We used the non-normalized data for multivariate analysis (MVA) and univariate statistics (UVA). A center scaling was used for MVA using SIMCA 16.0.1 software (Umea, Sweden). Unsupervised principal component analysis (PCA) was used to verify the quality of analysis, detect outliers, and check sample patterns. A partial least squares discriminant analysis (PLS-DA) model and orthogonal OPLS-DA were carried out to discriminate variation between groups. Model aptitude and predictive ability were evaluated using the explained variance (R^2^) and predicted variance (Q^2^), respectively, provided by the software. For UVA, we used MATLAB R2015a software (Mathworks, Inc., Natick, MA, USA), and a non-parametric 1-way ANOVA test (Kruskal–Wallis) was used to obtain the significant signals, followed by the Benjamini–Hochberg post hoc correction (FDR false discovery rate) (q = 0.05). Additionally, pairwise comparisons were obtained using the Mann–Whitney U-test. The level of statistical significance was established with a 95% confidence interval (*p* < 0.05). Finally, the percentage change was calculated (%). For infectivity and NO production analysis, the differences were evaluated by Student *t*-test (GraphPad software version 7).

### 4.7. Pathway Enrichment Analysis

Aiming to understand which pathways were modulated during the macrophage infection with *L. amazonensis* and evaluate how the parasite arginase can impact them even more. The biological pathways involved in the metabolism of the 53 metabolites from *La*-WT and *La*-arg^−^ infected C57BL/6-macrophages differentially modulated in comparison with uninfected and the biological function were analyzed by enrichment analysis using MetaboAnalyst (Figure S5). The corresponding pathways were shown based on *p* values from the enrichment analysis (*y*-axis) and fold enrichment (*x*-axis). The top 10 most impacted pathways were colored in red, and measured as closely related to *Leishmania*-infection of C57BL/6-macrophage, including urea cycle, glycine, and serine metabolism, ammonia recycling, methionine metabolism, aspartate metabolism, glutamate metabolism, spermidine and spermine biosynthesis, methylhistidine metabolism, and glutathione metabolism, supporting the involvement of arginine metabolism during infection. As expected, we observed that the amino acid L-arginine pathways were the most impacted during the infection.

This information guides us to integrate the differential regulated metabolites from arginine metabolism with other pathways in *La*-WT infected macrophages versus *La*-arg^−^ infected macrophages, as shown in Figure 6. The higher levels of arginine correlate with increased production of citrulline and ornithine. Ornithine support increased levels of trypanothione via polyamines production. Putrescine and spermidine were converted into glutathionylspermidine, and subsequent in trypanothione disulfide increased in *La*-WT infected macrophages. Additionally, ornithine can be interconverted in proline, and glutamate, raising the glutathione disulfide, both in higher infected macrophages. The alteration in pipecolate and xanthine levels showed the influences of *Leishmania* infection in the lysine degradation and purine metabolism.

### 4.8. Ethics Statement

The experimental protocol was approved by the Comissão de Ética no Uso de Animais (CEUA-approval number CEUA-IB: 314/2018)) from the Institute of Bioscience of the University of São Paulo. This study was carried out according to the recommendations in the guide and policies for the care and use of laboratory animals of the Brazilian government (Lei Federal 11.794, de 08/10/2008).

## 5. Conclusions

In conclusion, the parasite arginase plays a role in the metabolism of C57BL/6 macrophages infected with *Leishmania*, involving the metabolism of arginine, proline, and glutamate, and polyamines biosynthesis. It can be implicated in redox balance of host and parasite cells by oxidization and reduction in glutathione and trypanothione, NO production, influencing in the microbicidal activity of macrophages and parasite survival. The extrapolation of in vitro infections data to in vivo models or natural infection by *Leishmania* is not simple, but our data can contribute to the understanding of the early infection changes in the metabolic profile leading to macrophages response to infection [45].

## Figures and Tables

**Table 1:** Metabolite content from the comparison of macrophages infected with *La*-arg^−^ compared to *La*-WT infection extracted from the one-way ANOVA comparison among these two groups plus the uninfected.

Name	Mass (Da)	RMT	%RSD (QC)	*p*-Value ^(a)^	*p* FDR	*p*-Value ^(b)^	% Change	*p* (corr)	VIP
Glycine	75.0330	0.72	8.24	4.47 × 10^−6^	2.23E-05	4.33 × 10^−5^	−41.30	−0.84	<1
Putrescine	88.1000	0.42	5.81	3.92 × 10^−6^	2.07E-05	2.17 × 10^−5^	−58.46	−0.80	<1
Alanine	89.0483	0.77	7.31	1.85 × 10^−5^	4.74E-05	1.01 × 10^−2^	−24.82	−0.62	2.81
4-aminobutanoic acid	103.0647	0.67	7.41	1.85 × 10^−5^	4.74E-05	1.01 × 10^−2^	57.39	0.57	<1
Serine	105.0429	0.85	6.10	7.46 × 10^−6^	2.76E-05	2.60 × 10^4^	−37.88	−0.79	<1
Proline	115.0634	0.92	7.42	5.14 × 10^−6^	2.34E-05	2.17 × 10^−5^	−73.83	−0.96	1.73
Valine	117.0792	0.85	6.73	1.26 × 10^−5^	3.70E-05	2.99 × 10^3^	−30.00	−0.69	2.06
Pipecolic acid	129.0789	0.87	6.71	4.98 × 10^−6^	2.34E-05	6.50 × 10^4^	117.06	0.67	4.05
Isoleucine/leucine	131.0945	0.87	6.51	3.92 × 10^−6^	2.07E-05	2.17 × 10^−5^	−56.42	−0.91	4.16
Asparagine	132.0528	0.89	6.82	5.74 × 10^−6^	2.50E-05	1.52 × 10^4^	−41.67	−0.78	<1
Ornithine	132.0896	0.60	6.67	1.04 × 10^−2^	1.80E-04	2.17 × 10^−5^	−92.47	−0.99	2.00
Aspartic acid	133.0374	0.97	6.46	2.10 × 10^−2^	2.88E-02	1.72 × 10^−2^	−19.58	−0.56	<1
Hypoxanthine	136.0385	1.01	5.95	1.69 × 10^−5^	4.56E-05	7.62 × 10^3^	−26.98	−0.61	<1
Glutamic acid	147.0532	0.92	5.46	6.34 × 10^−5^	1.17E-04	4.14 × 10^3^	−26.26	−0.66	3.45
Methionine	149.0512	0.91	3.94	1.13 × 10^−5^	3.54E-05	2.10 × 10^3^	−34.19	−0.75	<1
Xanthine	152.0271	1.72	12.19	1.53 × 10^−2^	2.15E-02	2.79 × 10^−2^	19.21	0.50	<1
Histidine	155.0696	0.64	6.61	3.92 × 10^−6^	2.07E-05	2.17 × 10^−5^	−59.90	−0.93	1.96
Imidazolelactic acid	156.0554	0.76	8.51	1.06 × 10^−5^	3.41E-05	7.62 × 10^−3^	−27.91	−0.61	<1
Phenylalanine	165.0808	0.93	6.50	8.17 × 10^−6^	2.92E-05	6.50 × 10^−4^	−36.86	−0.75	1.05
1-Methylhistidine	169.0856	0.66	10.42	2.32 × 10^−6^	1.77E-05	2.17 × 10^−5^	−81.52	−0.98	<1
Arginine	174.1117	0.63	6.37	9.14 × 10^−6^	3.05E-05	9.74 × 10^−4^	100.48	0.66	3.77
Citrulline	175.0972	0.94	6.14	2.32 × 10^−6^	1.77E-05	2.17 × 10^−5^	220.70	0.82	<1
Argininic acid	175.0975	0.79	5.67	2.32 × 10^−6^	1.77E-05	2.17 × 10^−5^	1919.49	0.86	<1
Tyrosine	181.0739	0.96	5.81	1.26 × 10^−5^	3.70E-05	2.99 × 10^−3^	−37.73	−0.78	<1
Tryptophan	204.0894	0.93	5.11	1.54 × 10^−6^	1.77E-05	2.17 × 10^−5^	−27.53	−0.70	<1
*N*-carbamyl-arginine ^(c)^	217.1191	0.84	22.26	2.65 × 10^−6^	1.77E-05	4.33 × 10^−5^	*La*-WT ^(d)^	<0.5	<1
*N*-glutamyl-alanine ^(c)^	218.0906	1.03	7.04	2.32 × 10^−6^	1.77E-05	2.17 × 10^−5^	−51.06	−0.86	<1
Cystathionine	222.0679	0.85	4.06	6.61 × 10^−6^	2.64E-05	1.52 × 10^−4^	−57.72	−0.93	<1
Neuraminic acid ^(c)^	267.0961	1.13	7.88	2.65 × 10^−6^	1.77E-05	4.33 × 10^−5^	−40.89	−0.79	<1
*N*-(1-Deoxy-1-fructosyl)threonine ^(c)^	281.1118	1.13	7.78	9.14 × 10^−6^	3.05E-05	9.74 × 10^−4^	−44.93	−0.86	<1
Glutamylarginine ^(c)^	303.1550	0.76	9.35	6.27 × 10^−6^	2.61E-05	1.45 × 10^−3^	*La*-WT ^(d)^	<0.5	<1
S-Adenosylmethionine ^(c)^	398.1385	0.63	4.84	2.65 × 10^−6^	1.71E-05	4.33 × 10^−5^	−33.16	−0.74	<1
Trypanothione disulfide	721.2896	0.82	25.13	3.92 × 10^−6^	2.07E-05	2.17 × 10^−5^	−35.56	−0.71	<1
Trypanothione	723.3057	0.84	25.12	1.85 × 10^−5^	4.74E-05	1.01 × 10^−2^	−52.10	−0.80	<1

^(a)^ ANOVA *p*-values Kruskal Wallis test, ^(b)^ *p*–values Mann–Whitney U test, ^(c)^ tentative identification using only monoisotopic mass, and ^(d)^ metabolite present only in *La*-WT group. RMT: Relative migration time.

## Data Availability

Data is contained within the article or supplementary material.

## Supplementary Materials

The following are available online at https://www.mdpi.com/article/10.3390/ijms22136883/s1.

## Abbreviations

NOS2 nitric oxide synthase 2, NO nitric oxide, CAT2 cationic amino acid transporters 2, ARG1 arginase 1, AAP3 amino acid permease 3, *La*-WT *Leishmania amazonensis*-wild type, *La*-arg^−^ *Leishmania amazonensis*-arginase knockout, CE-MS Capillary Eletrophoresis-Mass Spectrometer, BMDMs bone marrow-derived macrophages.
