# Supplementary material for: Metabolomic Reprogramming of C57BL/6-Macrophages during Early Infection with L. amazonensis
Source: Int J Mol Sci. 2021 Jun 26;22(13):6883. doi: 10.3390/ijms22136883 (PMC8267886; doi:10.3390/ijms22136883)
Supplement: Supplementary file 1 [file ijms-22-06883-s001.zip › ijms-1244675-supplementary.pdf]

International Journal of *Molecular Sciences*

MDPI

Article

Metabolomic reprogramming of C57BL/6-macrophages during early infection with *L. amazonensis*

Supplementary Information (SI)

Supplementary Text for Experimental Section

Macrophage culture

Parasite culture

Metabolite Extraction

Supplementary Figures S1-S5

Figure S1. Infectivity of *La*-WT and *La*-arg- in C57BL/6-BMDMs macrophages.

Figure S2. Infectivity Analysis Gate Strategy.

Figure S3. Nitric Oxide Quantification Gates Strategy.

Figure S4. Scatter plot by Principal Component Analysis (PCA).

Figure S5. Metabolic pathways associated with metabolite enrichment in *L. amazonensis* infected macrophages.

Supplementary Table

Table S1. Metabolite content differentially identification in the comparison of C57BL/6-*La*-arg^{-}-infected or C57BL/6-*La*-WT-infected macrophages and uninfected macrophages

Supplementary Text for experimental Section

Macrophage culture

C57BL/6 6–8-week-old female mice were obtained from the Animal Center of the Faculty of Medicine of the University of São Paulo and maintained in the Animal Center of the Department of Physiology at the Institute of Bioscience of the University of São Paulo.

Mice bones were harvested, cut at each end, and flushed with a 23-gauge needle (BD, USA), removing the bone marrow with cold PBS to generate bone marrow-derived macrophages (BMDMs). Then, the cells were collected by centrifugation at 500 x *g* for 10 min at 4 °C, and resuspended in RPMI 1640 medium (LCBiotecnologia, São LGC, SP, Brazil), supplemented with penicillin (100 U/ml) (Invitrogen), streptomycin (100 μg/ml) (Invitrogen), 5% heat-inactivated FBS (Invitrogen), and 10% FBS9 cell supernatant into Vented-cap Culture Flask 75 cm^2 (SPL Lifescience, Pocheon, Ko). The cells were submitted to differentiation for 7–8 days at 34 °C in an atmosphere of 5% CO_2. The macrophage differentiation was confirmed by phenotypic analysis using flow cytometry (FACScalibur-Becton Dickinson, San Jose, CA, USA, considering 95% $F4/80^+/CD11b^+$ cells, as previously described.[1]

Parasite culture

L. amazonensis (MHOM/BR/1973/M2269) wild type promastigotes (*La*-WT) were maintained in culture at 25 °C in M199 medium (Invitrogen, Grand Island, NY, USA supplemented with 10% heat-inactivated fetal bovine serum (Invitrogen), 5 mg/L hemin, 100 μM adenine, 100 U penicillin, 100 μg/mL streptomycin, 40 mM Hepes-NaOH, and 12 mM $NaHCO_3$, at pH 6.85, for a week-long culture at low passage (P1-5). *L. amazonensis* arginase knockout (*La*-arg-) promastigotes were maintained in the same conditions as previously described, with medium supplemented with 30 μg/mL hygromycin B, 30 μg/mL puromycin (Sigma, St. Louis, MO, USA and 50 μM putrescine (Sigma).[2]

Metabolite Extraction

Pellets with $5x10^6$ uninfected or infected macrophages were resuspended in 350 μL of cold methanol/water (3:1, v:v) and 25 mg glass beads (710–1180 μM, G1152, Sigma Aldrich, Germany), followed by four cycles of frost/defrosting in a liquid N_2/37 °C bath. The cells were disrupted at 50 mHz for 10 min in TissueLyser LT (Qiagen, Germany). The samples were clarified by 15,700 x *g* for 10 min at 4 °C centrifugation, and the supernatant was collected and evaporated to dryness by SpeedVac SPD121P (Thermo Fisher Scientific, Waltham, MA) at 35 °C for 2 h. After this, the solid residue was resuspended in 100 μL 0.1 M formic acid with 0.2 mM of methionine sulfone, homogenized for 15 min in a vortex, and centrifuged at 15,700 x *g* for 15 min at 4 ºC. The 35 μL of supernatant was transferred to polypropylene vials (Agilent Techno Vials, Waldbronn, Germany) for analysis.[1] Quality-control (QC samples were prepared by pooling equal volumes of QC samples and were analyzed along the analytical sequence to evaluate the instruments stability and performance during measurements.

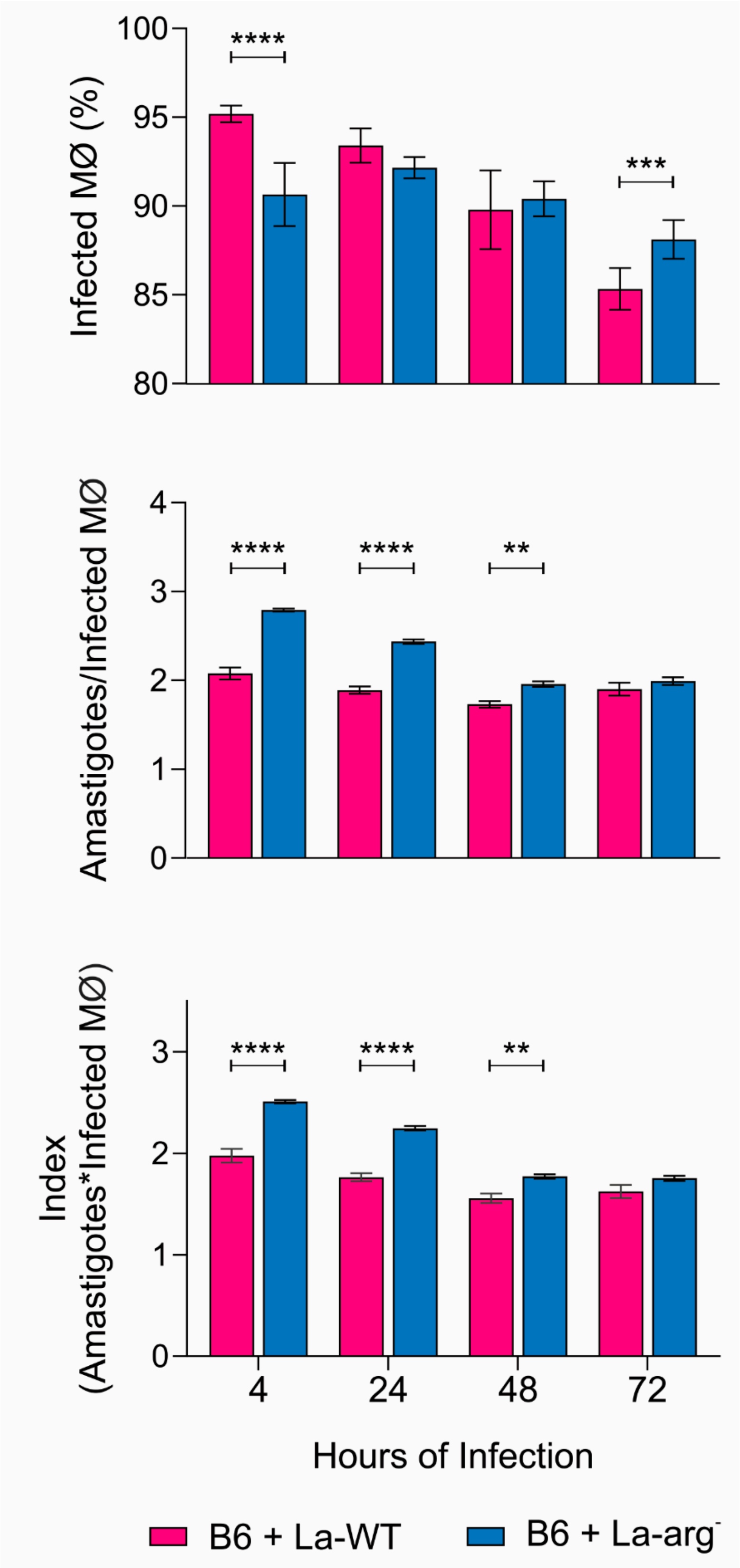

Figure S1. Infectivity of *La*-WT and *La*-arg- in C57BL/6-BMDMs macrophages. C57BL/6-macrophages (5x10^5) were infected with CFSE-labelled *L. amazonensis* (MOI5:1) and collected after 4, 24, 48, and 72 h for analysis of infectivity by image flow cytometer, gating in the CFSE-internalized cells to count the frequency of infected macrophages (infected MØ) and spot count tool to determine the number of infected MØ; index of infection was calculated by multiplying the rate of infected MØ by the number of amastigotes per infected MØ. Each bar represents the average and standard error of the mean (SEM) of values (n=6). (**) *p*-value < 0.01; (***) *p*-value < 0.001; (****) *p-value* < 0.0001.

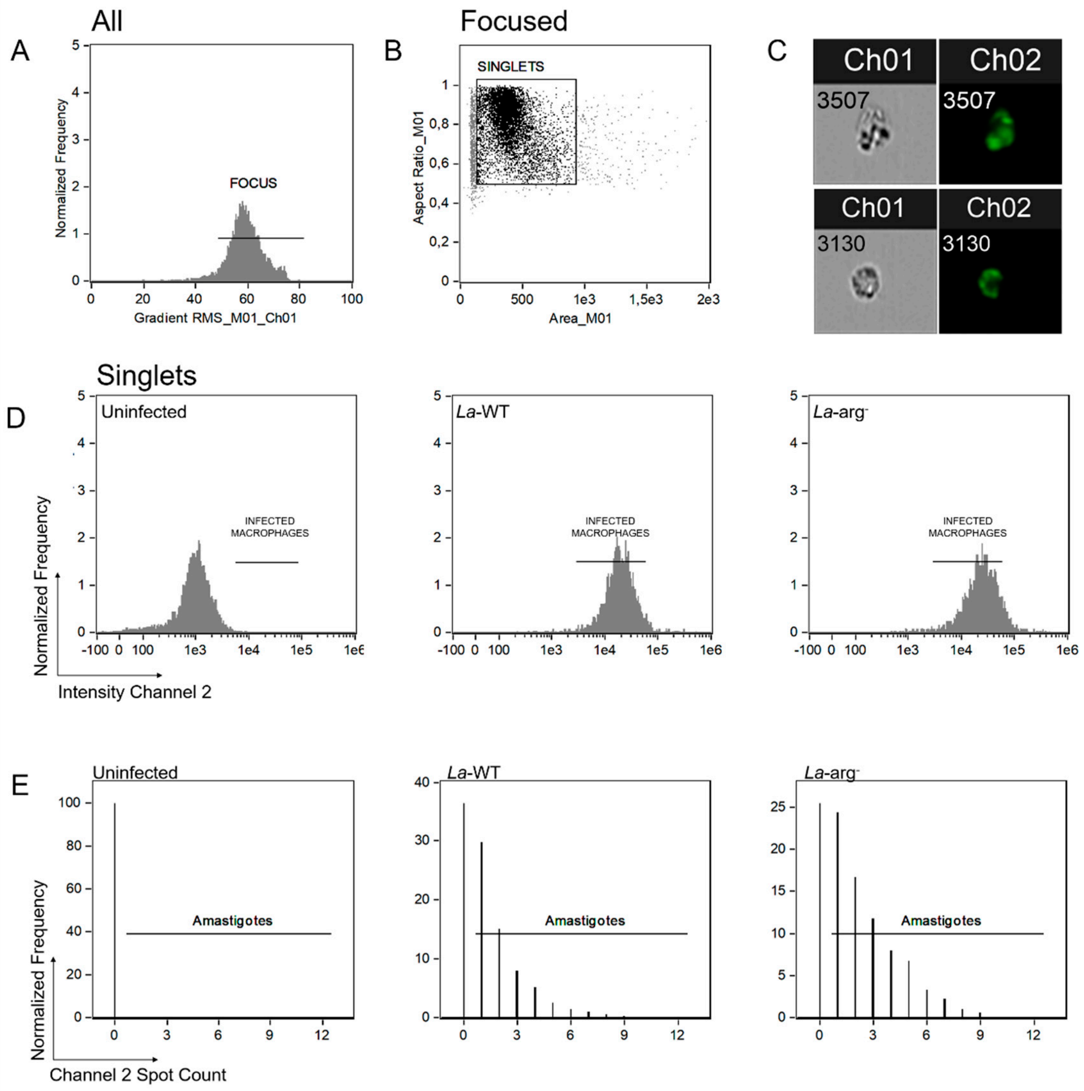

Figure S2. Infectivity analysis gate strategy. C57BL/6 bone marrow-derived macrophages were infected with CFSE labelled *L. amazonensis* (MOI 5:1) and had the infection curse monitored after 4, 24, 48 and 72 h; through image flow cytometry (Amnis FlowSight). The current thread was made using 4 h of infection as a model. Ten thousand events were acquired and then gated into (**A**) Camera focus. (**B**) The focused cells were then gated again to separate single cells from cellular debris or agglutinated cells. (**C**) The images are representative of brightfield (Channel 01 – 430-480 nm) and green channel (Channel 02 – 505-560 nm), where the green amastigotes are visible. (**D**) CFSE+ gate. (**E**) The number of amastigotes inside the infected macrophages was determined using the spot count feature. The distribution reveals the amastigote number counted inside macrophages.

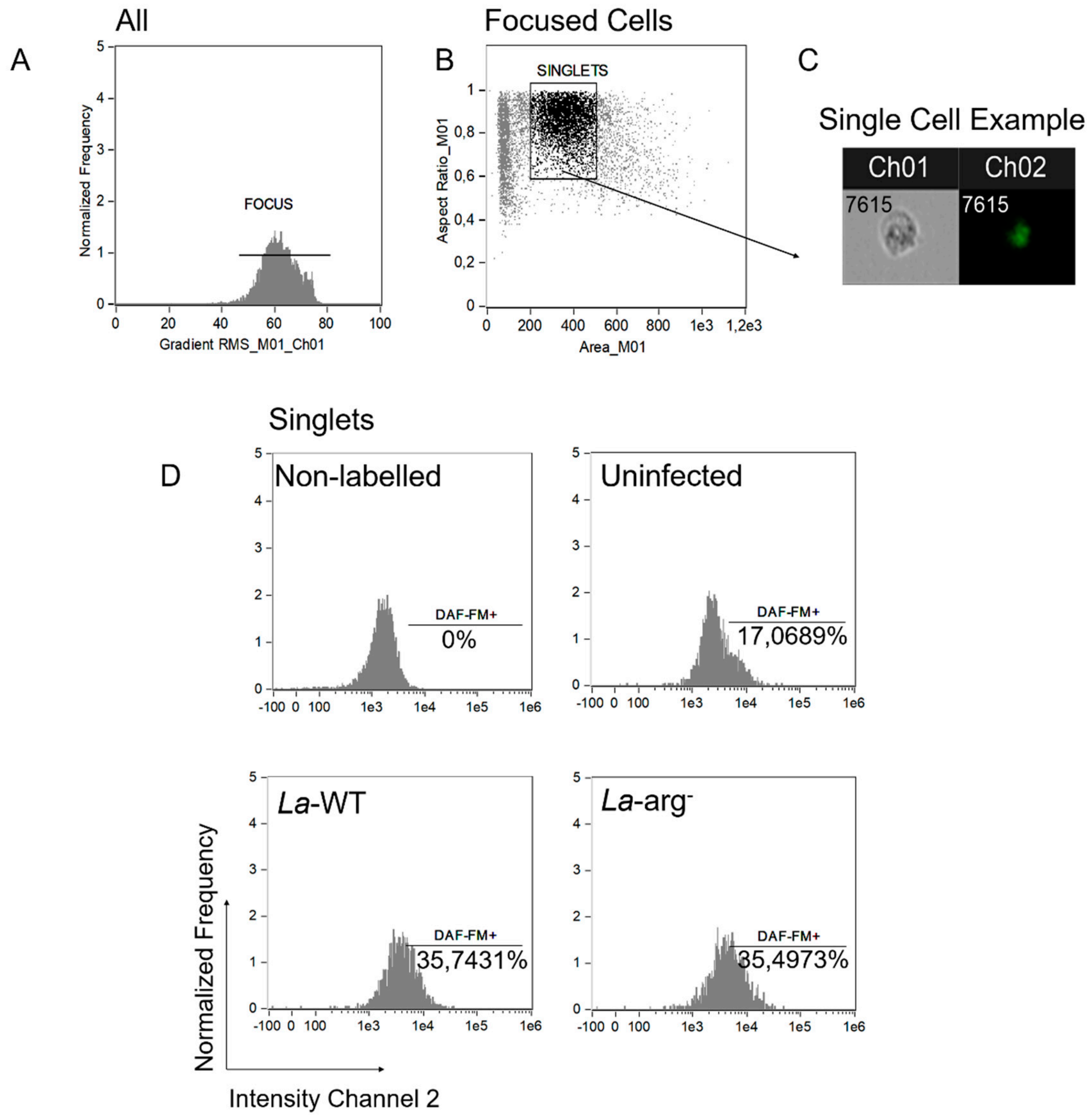

Figure S3. Nitric Oxide Quantification Gates Strategy. C57BL/6 bone marrow-derived macrophages were infected with *L. amazonensis* (MOI 5:1). After 4 h of infection, infected macrophages were treated with DAF-FM (5 μM – 30 ‘) and analyzed at image flow cytometer FlowSight (Amnis). (**A**) Ten thousand events were acquired and then gated into camera focus. (**B**) Focused cells were then gated into singlets, sorting from cellular debris or agglutinated cells. (**C**) Example of a single cell from singlets gate. (**D**) Distribution of DAF-FM$^+$ - gate made based on non-labelled control.

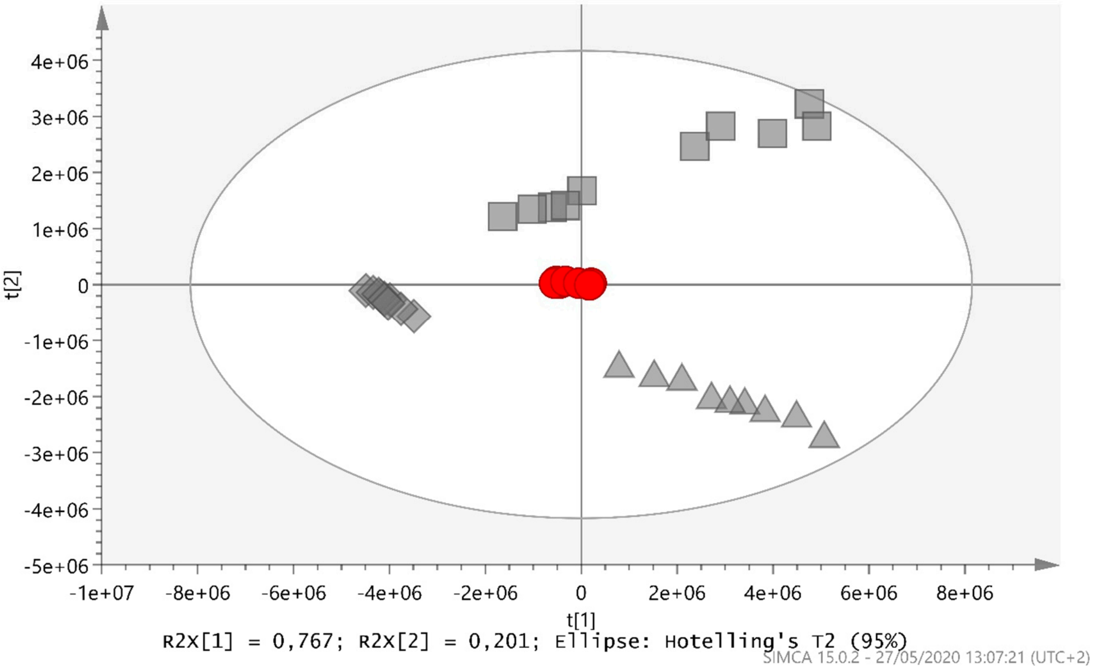

Figure S4. Scatter plot by Principal Component Analysis (PCA). PCA-X score plot with an explained variance (R^2) of 0.991, using non-normalized data and center scaling. C57BL/6-*La*-WT-infected macrophages (gray squares), C57BL/6-*La*-arg$^-$-infected macrophages (gray triangle), uninfected macrophages (gray diamond) and quality control (QC, red circle).

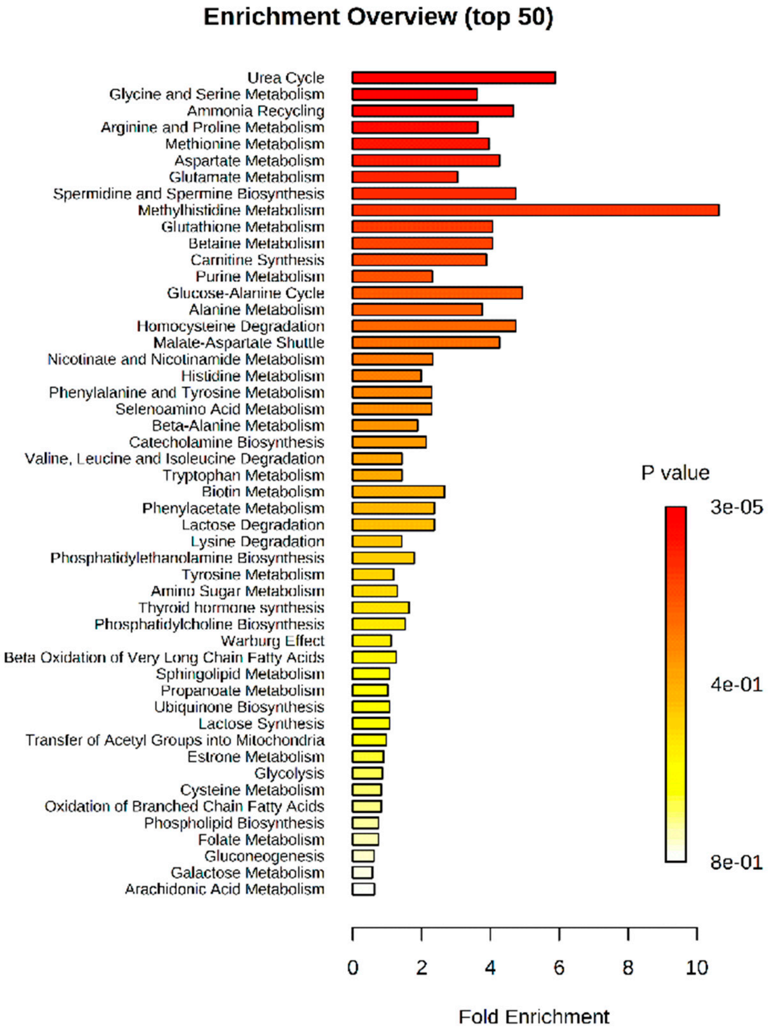

Figure S5. Metabolic pathways associated with metabolite enrichment in *L. amazonensis* infected macrophages. Analysis of dysregulated pathways based in metabolite peak areas from macrophages infected with *La*-WT or *La*-arg-, comparing to the uninfected one. We used the hypergeometric test to evaluate a particular metabolite set. The red color represents the maximum *p*-value, and the white color the minimum *p*-value.

Table S1. Metabolite content differentially identification in the comparison of C57BL/6-*La*-arg$^-$-infected or C57BL/6-*La*-WT-infected macrophages and uninfected macrophages.

						C57BL/6-*La*-WT vs. uninfected				C57BL/6-*La*-arg$^-$ vs. uninfected			
**Name**	**Mass (Da)**	**RMT**	**%RSD (QC)**	***p*-value[(a)]**	***p* FDR**	***p*-value[(b)]**	**%Change**	***p*(corr)**	**VIP**	***p*-value[(b)]**	**%Change**	***p*(corr)**	**VIP**
Glycine	75.0330	0.72	8.24	4.47E-06	2.23E-05	2.17E-05	549.9	0.98	<1	1.08E-05	281.5	0.87	<1
Putrescine	88.1000	0.42	5.81	3.92E-06	2.07E-05	2.17E-05	2287.5	0.91	<1	1.08E-05	891.8	0.94	<1
Alanine	89.0483	0.77	7.31	1.85E-05	4.74E-05	2.17E-05	2541.2	0.99	4.75	1.08E-05	1885.6	0.92	3.81
4-aminobutanoic acid	103.0647	0.67	7.41	1.85E-05	4.74E-05	2.17E-05	220.2	0.96	<1	1.08E-05	404.0	0.90	<1
Serine	105.0429	0.85	6.1	7.46E-06	2.76E-05	2.17E-05	291.8	0.97	<1	2.17E-05	143.4	0.82	<1
Proline	115.0634	0.92	7.42	5.14E-06	2.34E-05	2.17E-05	741.6	0.98	<1	4.33E-05	120.2	0.79	<1
Valine	117.0792	0.85	6.73	1.26E-05	3.70E-05	2.17E-05	1863.9	0.99	3.17	1.08E-05	1274.8	0.92	2.33
Betaine	117.0794	0.96	8.49	5.63E-05	1.06E-04	2.17E-05	321.1	0.89	<1	1.08E-05	421.5	0.93	<1
Niacinamide	122.0490	0.65	4.94	1.95E-02	2.71E-02	5.67E-03	33.4	0.64	<1	NS	NS	NS	NS
Pipecolic acid	129.0789	0.87	6.71	4.98E-06	2.34E-05	2.17E-05	*La*-WT[(d)]	0.99	2.36	1.08E-05	*La*-arg$^-$[(d)]	0.93	5.60
Itaconic acid [(c)]	130.0266	1.79	5.24	5.39E-05	9.81E-05	2.17E-05	-51.1	-0.92	<1	1.08E-05	-55.76	-0.91	<1
trans-4-hydroxyproline	131.0586	1.01	6.18	6.82E-05	1.24E-04	2.17E-05	1200.2	0.99	<1	1.08E-05	1660.1	0.92	1.06
Isoleucine/Leucine	131.0945	0.87	6.51	3.92E-06	2.07E-05	2.17E-05	644.5	0.98	4.03	1.08E-05	224.5	0.85	1.57
Asparagine	132.0528	0.89	6.82	5.74E-06	2.50E-05	2.17E-05	564.9	0.96	<1	1.08E-05	287.8	0.88	<1
Ornithine	132.0896	0.60	6.67	1.04E-04	1.80E-04	2.17E-05	1433.8	0.99	1.38	NS	NS	NS	NS
Aspartic acid	133.0374	0.97	6.46	2.10E-02	2.88E-02	2.20E-02	16.6	0.55	<1	NS	NS	NS	NS
Adenine	135.0558	0.67	25.43	2.40E-05	5.58E-05	2.17E-05	*La*-WT[(d)]	0.94	<1	1.08E-05	*La*-arg$^-$[(d)]	0.92	<1
Hypoxanthine	136.0385	1.01	5.95	1.69E-05	4.56E-05	2.17E-05	620.7	0.96	1.22	1.08E-05	426.3	0.87	<1
4-Guanidinobutyric acid	145.0875	0.72	8.99	3.19E-05	7.17E-05	2.17E-05	614.7	0.96	<1	1.08E-05	457.5	0.85	<1
Acetylcholine	145.1118	0.70	19.85	1.23E-02	1.81E-02	1.01E-02	37.3	0.61	<1	9.45E-03	52.2	0.58	<1
Glutamine	146.0690	0.91	2.68	7.31E-05	1.28E-04	2.17E-05	172.9	0.93	<1	1.08E-05	197.5	0.88	<1

(a) *p*-values Kruskal Wallis test, [(b)] *p* –values Mann-Whitney U test, [(c)] Tentative identification using only monoisotopic mass, [(d)] metabolite present only in one group in the comparison to C57BL/6-*La*-WT or C57BL/6-La-arg$^-$ infected to samples uninfected, RMT: Relative migration time and NS: non-significative.

Table S1 cont. Metabolite content differentially identification in the comparison of C57BL/6-*La*-arg^{-}-infected or C57BL/6-*La*-WT-infected macrophages and uninfected macrophages

						C57BL/6-*La*-WT vs. uninfected				C57BL/6-*La*-arg vs. uninfected			
**Name**	**Mass (Da)**	**RMT**	**%RSD (QC)**	***p*-value[a]**	***p* FDR**	***p*-value[b]**	**%Change**	***p*(corr)**	**VIP**	***p*-value[b]**	**%Change**	***p*(corr)**	**VIP**
Lysine	146.1055	0.61	6.66	3.47E-05	7.53E-05	2.17E-05	481.3	0.98	1.22	1.08E-05	390.5	0.90	1.06
Glutamic acid	147.0532	0.92	5.46	6.34E-05	1.17E-04	2.17E-05	123.7	0.93	3.63	2.09E-03	64.9	0.66	2.26
Methionine	149.0512	0.91	3.94	1.13E-05	3.54E-05	2.17E-05	707.3	0.98	<1	1.08E-05	431.3	0.89	<1
Xanthine	152.0271	1.72	12.19	1.53E-02	2.15E-02	NS	NS	NS	NS	5.20E-03	24.51	0.50	<1
Histidine	155.0696	0.64	6.61	3.92E-06	2.07E-05	2.17E-05	2406.4	0.99	1.99	1.08E-05	905.0	0.92	<1
Imidazolelactic acid	156.0554	0.76	8.51	1.06E-05	3.41E-05	2.17E-05	*La*-WT[d]	0.99	<1	1.08E-05	*La*-arg^{-}[d]	0.91	<1
Phenylalanine	165.0808	0.93	6.5	8.17E-06	2.92E-05	2.17E-05	1643.0	0.98	1.43	1.08E-05	1000.5	0.91	<1
1-Methylhistidine	169.0856	0.66	10.42	2.32E-06	1.77E-05	2.17E-05	*La*-WT[d]	0.99	<1	1.08E-05	*La*-arg^{-}[d]	0.94	<1
Arginine	174.1117	0.63	6.37	9.14E-06	3.05E-05	2.17E-05	312.2	0.98	2.06	1.08E-05	726.4	0.92	5.05
Citrulline	175.0972	0.94	6.14	2.32E-06	1.77E-05	2.17E-05	*La*-WT[d]	0.99	<1	1.08E-05	*La*-arg^{-}[d]	0.94	<1
Argininic acid	175.0975	0.79	5.67	2.32E-06	1.77E-05	2.17E-05	*La*-WT[d]	0.96	<1	1.08E-05	*La*-arg^{-}[d]	0.92	<1
Tyrosine	181.0739	0.96	5.81	7.29E-06	2.76E-05	2.17E-05	1085.6	0.99	<1	1.08E-05	638.3	0.91	<1
N-acetylspermidine [c]	187.1661	0.56	14.7	4.43E-05	9.42E-05	2.17E-05	*La*-WT[d]	0.79	<1	1.08E-05	*La*-arg^{-}[d]	0.92	<1
N_6, N_6, N_6-trimethyllysine	188.1507	0.63	5.21	3.23E-05	7.17E-05	2.17E-05	*La*-WT[d]	0.98	<1	1.08E-05	*La*-arg^{-}[d]	0.93	<1
Acetylcarnitine	203.1174	0.77	2.35	1.51E-02	2.15E-02	4.14E-03	31.9	0.66	<1	NS	NS	NS	NS
Glutamylglycine [c]	204.0745	1.01	4.69	4.97E-05	9.57E-05	2.17E-05	*La*-WT[d]	0.99	<1	1.08E-05	*La*-arg^{-}[d]	0.94	<1
Tryptophan	204.0894	0.93	5.11	1.26E-05	3.7E-05	2.17E-05	876.2	0.99	<1	1.08E-05	607.4	0.92	<1
N-carbamylarginine [c]	217.1191	0.84	22.26	1.54E-06	1.77E-05	2.17E-05	*La*-WT[d]	NS	NS	NS	NS	NS	NS
N-glutamylalanine [c]	218.0906	1.03	7.04	2.65E-06	1.77E-05	2.17E-05	*La*-WT[d]	0.99	<1	1.08E-05	*La*-arg^{-}[d]	0.93	<1
Cystathionine	222.0679	0.85	4.06	2.32E-06	1.77E-05	2.17E-05	*La*-WT[d]	0.99	1	1.08E-05	*La*-arg^{-}[d]	0.94	<1
Neuraminic acid [c]	267.0961	1.13	7.88	6.61E-06	2.64E-05	2.17E-05	279.5	0.97	<1	2.17E-05	124.3	0.80	<1

[a] *p*-values Kruskal Wallis test, [b] *p* –values Mann-Whitney U test, [c] Tentative identification using only monoisotopic mass, [d] metabolite present only in one group in the comparison to C57BL/6-*La*-WT or C57BL/6-La-arg^{-} infected to samples uninfected, RMT: Relative migration time and NS: non-significative.

Table S1 cont. Metabolite content differentially identification in the comparison of C57BL/6-*La*-arg^{-}-infected or C57BL/6-*La*-WT-infected macrophages and uninfected macrophages.

						C57BL/6-*La*-WT vs. uninfected				C57BL/6-*La*-arg vs. uninfected			
**Name**	**Mass (Da)**	**RMT**	**%RSD (QC)**	***p*-value**[(a)]	***p* FDR**	***p*-value**[(b)]	**%Change**	***p*(corr)**	**VIP**	***p*-value**[(b)]	**%Change**	***p*(corr)**	**VIP**
Deoxyguanosine	267.0968	1.03	23.03	4.74E-05	9.57E-05	2.17E-05	*La*-WT[(d)]	0.95	<1	1.08E-05	*La*-arg^{-}[(d)]	0.85	<1
Adenosine	267.0980	0.86	4.84	8.83E-03	1.38E-02	7.62E-03	149.4	0.53	<1	4.33E-02	41.6	<0.5	<1
Argininosuccinic acid [3]	272.1132	0.74	5.24	7.10E-05	1.27E-04	2.17E-05	6790.0	0.98	<1	1.08E-05	8015.5	0.89	<1
Glutamylglutamic acid [(c)]	276.0944	1.09	7.87	2.24E-05	5.34E-05	2.17E-05	*La*-WT[(d)]	0.99	<1	1.08E-05	*La*-arg^{-}[(d)]	0.94	<1
N-(1-Deoxy-1-fructosyl)threonine [(c)]	281.1118	1.13	7.78	2.65E-06	1.77E-05	2.17E-05	*La*-WT[(d)]	0.99	<1	1.08E-05	*La*-arg^{-}[(d)]	0.94	<1
Glutamylarginine [(c)]	303.155	0.76	9.35	1.54E-06	1.77E-05	2.17E-05	*La*-WT[(d)]	<0.5	<1	NS	NS	NS	NS
Aspartylglycosamine [(c)]	335.1332	1.08	6.63	4.89E-05	9.57E-05	2.17E-05	*La*-WT[(d)]	0.99	<1	1.08E-05	*La*-arg^{-}[(d)]	0.94	<1
S-adenosylmethionine [(c)]	398.1385	0.63	4.84	9.14E-06	3.05E-05	2.17E-05	665.2	0.99	<1	1.08E-05	411.4	0.89	<1
Oxidized glutathione	612.1536	1.02	28.87	1.14E-02	1.70E-02	1.45E-03	71.5	0.68	<1	4.33E-02	66.1	0.61	<1
Trypanothione disulfide	721.2896	0.82	25.13	6.27E-06	2.61E-05	2.17E-05	*La*-WT[(d)]	0.98	<1	1.08E-05	*La*-arg^{-}[(d)]	0.91	<1
Trypanothione	723.3057	0.84	25.12	2.65E-06	1.71E-05	2.17E-05	*La*-WT[(d)]	0.97	<1	1.08E-05	*La*-arg^{-}[(d)]	0.91	<1

[(a)] *p*-values Kruskal Wallis test, [(b)] *p* –values Mann-Whitney U test, [(c)] Tentative identification using only monoisotopic mass, [(d)] metabolite present only in one group in the comparison to C57BL/6-*La*-WT or C57BL/6-La-arg^{-} infected to samples uninfected, RMT: Relative migration time and NS: non-significative.

References:

1. Muxel, S. M.; Mamani-Huanca, M.; Aoki, J. I.; Zampieri, R. A.; Floeter-Winter, L. M.; López-González, Á.; Barbas, C., Metabolomic profile of BALB/c macrophages infected with *Leishmania amazonensis*: Deciphering l-arginine metabolism. *Int. J. Mol. Sci.* **2019,** *20* (24). DOI: 10.3390/ijms20246248.
2. da Silva, M. F.; Zampieri, R. A.; Muxel, S. M.; Beverley, S. M.; Floeter-Winter, L. M., *Leishmania amazonensis* arginase compartmentalization in the glycosome is important for parasite infectivity. *PLoS One* **2012,** *7* (3), e34022. DOI: 10.1371/journal.pone.0034022.
3. Mamani-Huanca, M.; Gradillas, A.; López-González, Á.; Barbas, C., Unraveling the Cyclization of l-Argininosuccinic Acid in Biological Samples: A Study via Mass Spectrometry and NMR Spectroscopy. *Anal Chem* **2020,** *92* (19), 12891-12899. DOI: 10.1021/acs.analchem.0c01420.
